# Comparison of mechanical energy transfer during right-forward lunge between female amateur and professional badminton players

**DOI:** 10.1186/s13102-023-00741-0

**Published:** 2023-09-28

**Authors:** Soheila Safavi, Rahman Sheikhhoseini, Sajjad Abdollahi

**Affiliations:** 1 Department of corrective exercise & Sport injury, Faculty of physical education and sport sciences, Allameh Tabataba’i University, western Bulverde of Azadi sport complex, Tehran, Iran; 2 Department of corrective exercise & Sport injury, Faculty of physical education and sport sciences, Allameh Tabataba’i University, Tehran, Iran

**Keywords:** Badminton Players, Power Flow, Mechanical energy transfer, Backhand Stroke

## Abstract

**Background:**

Regarding their skill levels, badminton players present different movement patterns during front and right lunging. The main objective of this study was to compare the mechanical energy transfers attributable to right-forward lunges between amateur and professional badminton players to study variations in mechanical efficiency at various skill levels.

**Method:**

In this cross-sectional study, twenty female badminton players were recruited (Professional group n = 10 and Amateur group n = 10). The kinematics and kinetics of the lower extremities were recorded while performing right-forward lunges using Vicon motion capture and Kistler force plates. Mechanical energy expenditures (MEE) were extracted in eccentric transfer, concentric transfer, and no-transfer phases for the hip, knee, and ankle joints. At each joint, mechanical energy compensations (MEC) were also determined. Independent samples t-tests were used to analyze data at a significance level of α = 0.05.

**Result:**

Regards to mechanical energy expenditures at the initial heel contact phase, the professional players demonstrated statistically significant more ankle no-transfer (p < 0.003), less knee concentric transfer (p < 0.026), more knee eccentric transfer (p < 0.001), and less hip no-transfer (p < 0.001). At the same time, the amateur athletes showed significantly more ankle eccentric transfer (p < 0.042) at maximal knee flexion angle time point. Analyzing mechanical energy compensation coefficients showed that the professional athletes had significantly less ankle concentric transfer (p < 0.001), more knee concentric transfer (p < 0.001), more knee eccentric transfer (p < 0.001), and more hip eccentric transfer (p < 0.001) at initial contact phase. While they found to have significantly more ankle eccentric transfer (p < 0.007), less knee concentric transfer (p < 0.001), less knee eccentric transfer (p < 0.001), more hip concentric transfer (p < 0.001), and more hip eccentric transfer (p < 0.001) at maximal knee flexion angle.

**Conclusion:**

it is shown that the mechanical energy efficiency of the right-forward lunge is skill-related. It seems that altered lunge landing biomechanics may increase the risk of ankle and knee injuries and muscular damages in amateur athletes. It is recommended for amateur players to follow a injury prevention training program that promotes proper lunging technique.

**Supplementary Information:**

The online version contains supplementary material available at 10.1186/s13102-023-00741-0.

## Introduction

Badminton is among the most popular racquet sports worldwide [1, 2]. It has been reported that badminton frequently result in joint injuries, which suggests that the joint loads during play may be extremely high [3]. Injury in the lower limb is the most common in badminton, with an increased injury risk when the level of playing skills increases [4, 5]. Ankles, knees, and hips have been recognized as the most prevalent locations of sport-related injuries like sprains, strains, and tears [6]. Furthermore, a previous study showed that the incidence of sport-related injuries might be different between professional and non-professional badminton players [6]. This may be due to different movement patterns performed while training by athletes.

In badminton, the lunge is a crucial move that allows players to quickly move into the ideal situation for the next shot, return to the starting position, or go off in another direction for the next movement [7–9]. The lunge accounted for more than 15% of all movements during a competitive singles match [9]. Badminton has a higher risk of injury than other sports due to the unbalanced loading patterns and impact stress placed on the ankle, knee, and hip joints during right-forward lunging step actions [7–12].

On the other hand, highly rated youth badminton players appear to perform a stroke technique differently compared to competitors with lower ranks [7–9, 12]. However, while viewing them in a training session, it can be challenging to pinpoint the precise differences in the execution. Recent findings showed different lunge biomechanics between amateur and professional badminton players [7]. While in comparison with professionals, amateur athletes typically land the right-forward lunging stride with larger ankle, knee, and hip angles [7, 12] and greater knee abduction moment [8, 9]. Also, it has shown that badminton players might adopt different biomechanics strategies to lunge in four directions [10, 13]. However, there is insufficient evidence to evaluate Mechanical Energy Transfer (MET) between segments or joints during motions.

METs are the capacity to plan appropriate interactions between joints or segments during motions to create an efficient, smooth, and accurate movement pattern while the person moves [14–16]. The movement coordination during motion might reflect neuromuscular synergies [17]. Moreover, Mechanical Energy Expenditure (MEE) is considered the efficiency of mobility and energy transfers. MEE is the sum of the Mechanical Energy Compensations (MEC), agonist, synergist, and antagonist activity at a joint, and the net amount of energy created by the muscles directing the movements [18]. In contrast, an inability to modulate MET may induce movement deviations [17, 19]. It seems that energy-transfer processes change with the level of skills and links to lower extremity injuries [1, 20]. These alterations are linked to worse balance control, decreased muscle mass and strength, and higher energy expenditure during actions like lunging [7, 10, 12, 16]. In this regard, a previous study showed different MET in the upper extremities of badminton athletes with different levels of skill [21]. However, the possible differences between MET in lower extremities remained unclear.

It appears logical to assume that the mentioned kinematic changes among athletes and players during right-forward lunging would result in varying mechanical efficiency by changing the amount of energy generated or absorbed by the muscles at the time of initial contact and peak knee flexion. However, it is unknown to what extent this occurs or whether specific energy flow deficits can be made up elsewhere. Understanding the energy costs connected to right-forward lunging in athletes and players requires determining MET and the muscular energy compensated by inter-segmental energy transfer. Therefore, this study aimed to compare the MET between professional and amateur badminton players during right-forward lunging.

## Methodology

### Participants

In this cross-sectional study, 20 female badminton players, including 10 high national-level players and 10 recreational college players, participated. The sample size was estimated using data from a previous study investigating plantar pressure differences among badminton players. It was discovered by utilizing G*Power ver. 3.1 A minimum of 20 individuals would be needed to reach 90% statistical power with an alpha level of 0.05 [12]. Right-handed female badminton players, ages 16 to 18 years, with at least five years’ history of playing badminton in Iran’s professional league, were recruited as professional athletes.

In comparison, female students in the same age range who played badminton recreationally were selected as amateur players. The exclusion criteria were: subjects with a history of any sport-related injuries to the lower and upper extremities in the past 6 months that limit participating in the sport at the time of the study, history of the previous ligament or capsular surgeries in the lower and upper extremities, history of surgery in the spinal column, present using of any medicine for medical conditions, and presence of pain or discomfort while performing the study protocol in any segments of the body. Before starting the investigation, methodological approval was obtained from the Biomedical Research Ethics Committee of the University of Social Welfare and Rehabilitation Sciences (Ethics code: IR.USWR.REC.1397.093). Before the study started, all participants were informed in writing and orally about the study procedures during a familiarization session. Each participant in this session signed an informed consent form.

### Experimental design

The subjects’ demographic information, such as age, gender, height, and weight, was gathered before the studies. In order to set up the appropriate experimental setting for each subject, measurements of the height of the Anterior Superior Iliac Spine (ASIS) and leg length were also calculated. When doing lunge tasks, the individuals wore their badminton shoes. Kinematics and kinetics data were constantly gathered throughout each lunge motion during the study sessions. To determine the joint center and joint neutral position, two trials in static standing were conducted before the right-forward lunge trials. In addition, before beginning the lunge trials, the participants were asked to warm up for five minutes.

**Fig. 1:**
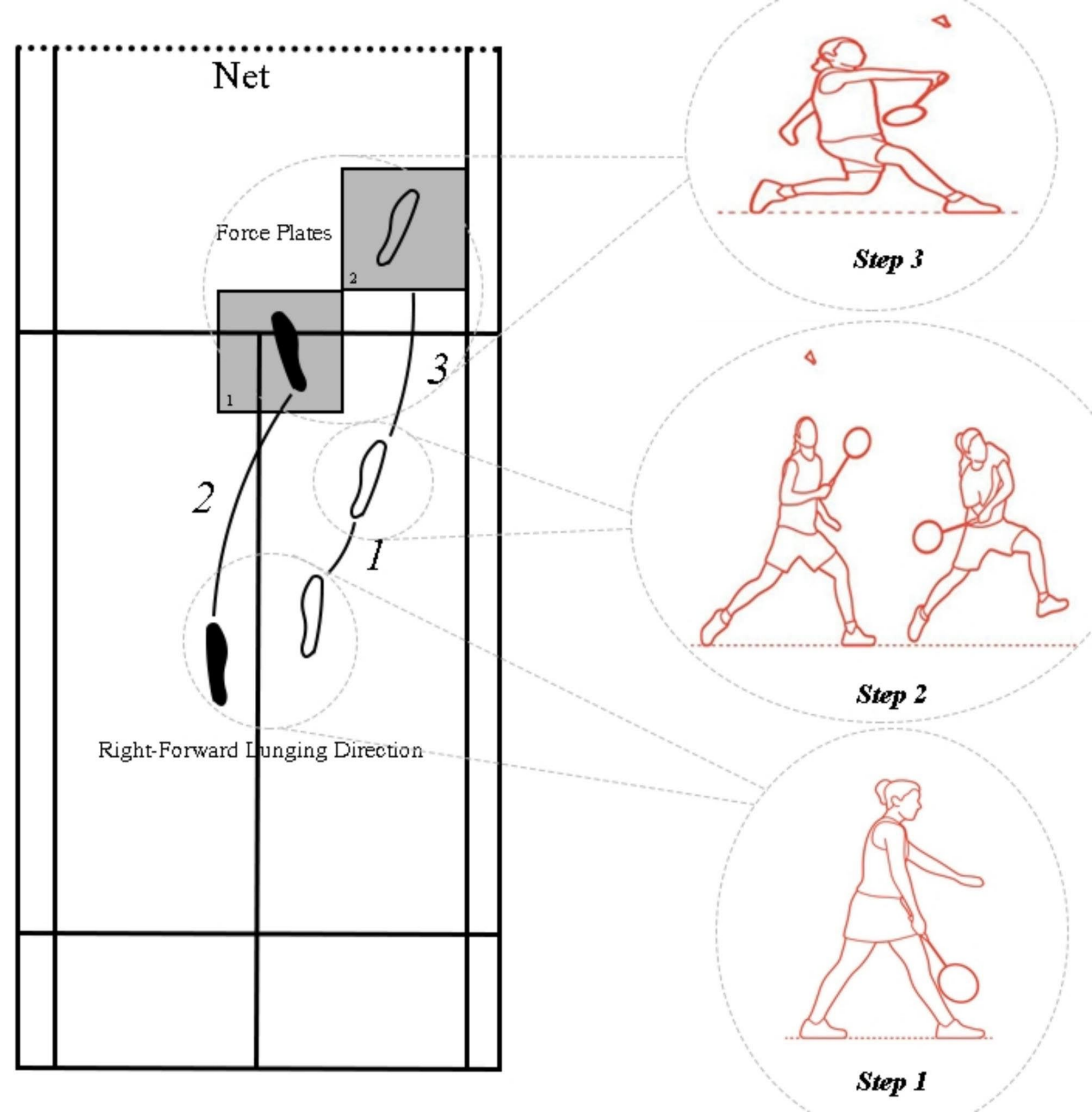
Illustration of right-forward lunge direction. Open foot marks indicate dominant foot positions, whereas solid foot marks indicate non-dominant foot placements. The numerals represent the steps on the force plates

The present study focuses on the lunge movement because prior research has shown that repeatedly performing rapid lunges that involve strenuous impact during the heel contact phase can place excessive stress on the lower extremities of players. This, in turn, can increase the risk of lower extremity injuries among the players [8, 9]. During the test, the examiner directed the players to begin their lunge movement while her racket was placed along the left side. They were told to straighten their right knee and land on a force plate while hitting a suspended shuttlecock with a backhand shot. This was meant to simulate a situation in a badminton game where the shuttlecock is dropped in the front court. The players were instructed to use a backhand grip, with their thumb pointing upwards, to hit the shuttlecock in front of their body and execute a backhand shot by lifting their shoulder and upper limb (see Fig. 1) [22]. All participants completed five successful forward lunge trials with backhand shots, with a 30- to 60-second break in between. Each right-forward lunge task required the participants to push off the dominant lower extremity and return to the standing position after running two steps on the non-dominant lower extremity in an anterior direction. The participants were asked to step on the force plate with the dominant leg and hit the shuttlecock [23].

Additionally, the shuttlecock was hanged at the height of each participant’s ASIS for each trial. Before beginning the lunge movements, the participants had enough time to practice up to five times, so they were comfortable with the necessary movement pattern and the testing set. Each participant was asked to perform the right-forward lunge movement for three trials. Trials were deemed great if the subject’s dominant limb got in touch with the force plate’s center, the shuttlecock was touched, and the subject could go back to the initial posture from the lunge (see Fig. 1). The relevant factors in these tests were averaged together to get a mean value. A biomechanist who was not aware of the respondents’ group designations oversaw the execution of all of the information-gathering procedures.

### Data analysis

**Fig. 2:**
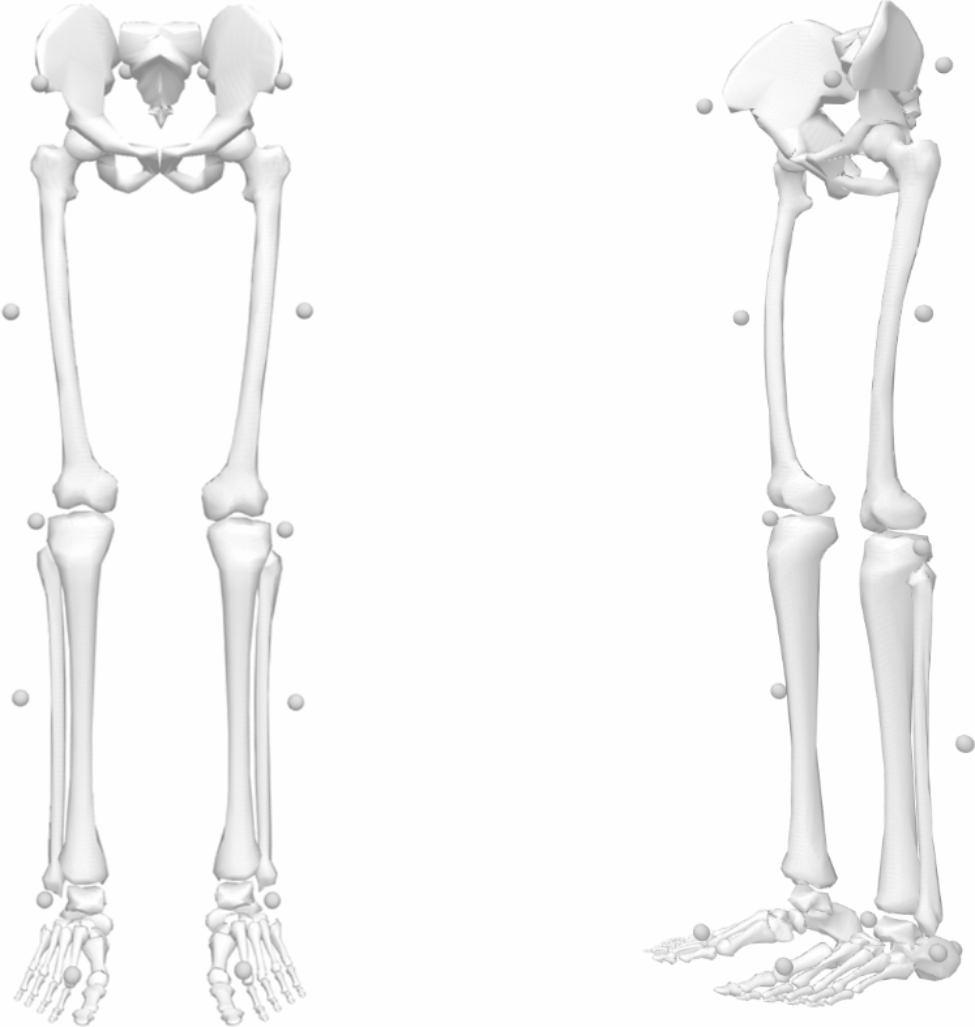
Marker placement based on the Plug-in-Gait Model for motion analyses

16 reflective markers (diameter of 14 mm) were used to pinpoint the precise locations of the lower limb segments locally. The marker locations included ASIS, lateral tibia (TIB), lateral knee (KNE), lateral ankle (ANK), posterior-superior iliac spine (PSI), lateral thigh (THI), heel (HEE) and toe (TOE) to the left and right lower extremity (Fig. 2). The same investigator placed the markers for all participants. Each subject completed two static standing trials before beginning the lunge exercises. During the lunge tasks, the trajectories of the 16 markers were recorded using an eight-camera high-speed motion analysis system (Motion Analysis Corporation, Santa Rosa, CA, USA) at a rate of 330 frames s–1. The ground reaction force was acquired using two synchronized Kistler force plates (Type 9286Ba, Kistler Inc., Winterhur, Switzerland) at a sampling rate of 1200 Hz.

The investigation concurrently recorded ground reaction forces and kinematic data. For the study, each respondent’s kinematic and kinetic data were averaged over three successful attempts. Each body segment’s three-dimensional rotations and translations were obtained by filtering the information using a MATLAB program and a Butterworth low-pass filter with a fourth-order and a 6 Hz cut-off [24]. Regression equations [25] were used to calculate segmental body mass, the center of mass, and the moment of inertia of mass movement based on individual subjects’ anthropometric data. By numerically differentiating segment position information, segment both angular and linear speeds and accelerations were calculated. Then, these values were combined with segment mass-inertial information to calculate the net joint torques using the Newtonian inverse dynamic technique [26, 27].

### Mechanical energy transfer calculations

In the case of circular energy, the muscle moment (${M}_{j}$where ${M}_{j}^{p}$= ${-M}_{j}^{d}$) and the segment’s angular speed (ω) were combined to determine the power of the proximal (${P}_{p}$) and distal (${P}_{d}$) ends of the distal and proximal articulating segments, accordingly. This power was referred to as the proximal and distal powers, respectively. At a specific joint (*j*), the net muscle power (${P}_{j}$, W/kg) is the sum of the powers at the proximal ends of the preceding and subsequent segments:

$${P_j} = M_j^p({\omega ^p} - {\omega ^d}) = M_j^p{\omega ^p} + M_j^d{\omega ^d} = {P_p} + {P_d}$$

Where M is muscle moment, (N.m/kg), v represents angular velocity (rads/s), and P is muscle power (W/kg). Whether the muscles generate or consume energy and whether or not energy is transmitted between muscle fibers can be deduced from the signature and relative amplitude of the power terms [18, 28–30].

In order to determine the total energy generated by the system, the mechanical energy expenditure (MEE) was calculated. This was done by using the methodology that McGibbon and colleagues provided [18, 29, 30].

$${U}_{j}={\int }_{{t}_{1}}^{{t}_{2}}\left|{P}_{j}\right|dt$$

MEC was calculated as the difference between the net MEE at the joint and the absolute MEE at the joint using Aleshinsky’s method [31].

$${G}_{j}=1-\frac{{\int }_{{t}_{1}}^{{t}_{2}}\left|{P}_{j}\right|dt}{{\int }_{{t}_{1}}^{{t}_{2}}\left[\left|{P}_{d}\right|+\left|{P}_{p}\right|\right]dt}$$

Concentric transfer (MEEC = MEEcondition 3 + MEEcondition 5), eccentric transfer (MEEE = MEEcondition 4 + MEEcondition 6), and no transfer conditions (MEEN = - MEEcondition 1 + MEEcondition 2) were calculated independently for the overall MEE of the pair. The MEE was calculated using the average of the net joint power curve after dividing it into power conditions. MEC, also known as muscular power compensation, or MEC, is the proportion of muscular power offset by inter-segmental energy conversion in addition to the net joint MEE [18], which was determined for the number of both concentric and eccentric actions that each joint underwent. The MEC was found by subtracting the absolute and complete MEE from the net joint MEE for each type of compression to arrive at the correct answer (concentric, eccentric). MEC generally equals zero in the absence of a segmental transfer.

### Statistical analysis

Descriptive statistics (means and standard deviations) were computed for every metric used to characterize the sample’s demographics and outcomes. Independent samples t-tests were used to examine the MEE and MEC measures at the ankle, knee, and hip to distinguish between groups. For each analysis, the meaningful threshold was set at 0.05. All studies were carried out using SPSS software version 24.0 (SPSS, Chicago, IL, USA).

## Results

Table 1 displays the demographic information for the people who were enrolled. Age, body mass, height, and BMI were not significantly different between the two groups.

**Table 1:** Participant demographics

Variable	Professionals (N = 10)	Amateurs (N = 10)	P-value
	Mean ± SD	Mean ± SD	
Age (years)	20.1 ± 0.9	18.0 ± 1.6	0.250
Height(cm)	165.2 ± 5.4	164.4 ± 6.4	0.769
Body Mass (kg)	58.0 ± 2.6	55.7 ± 5.5	0.256
BMI (kg.m2)	21.3 ± 1.5	20.6 ± 2.4	0.507

**Note**: *P-value < 0.05 is considered to be statistically significant. BMI, Body Mass Index

**Fig. 3:**
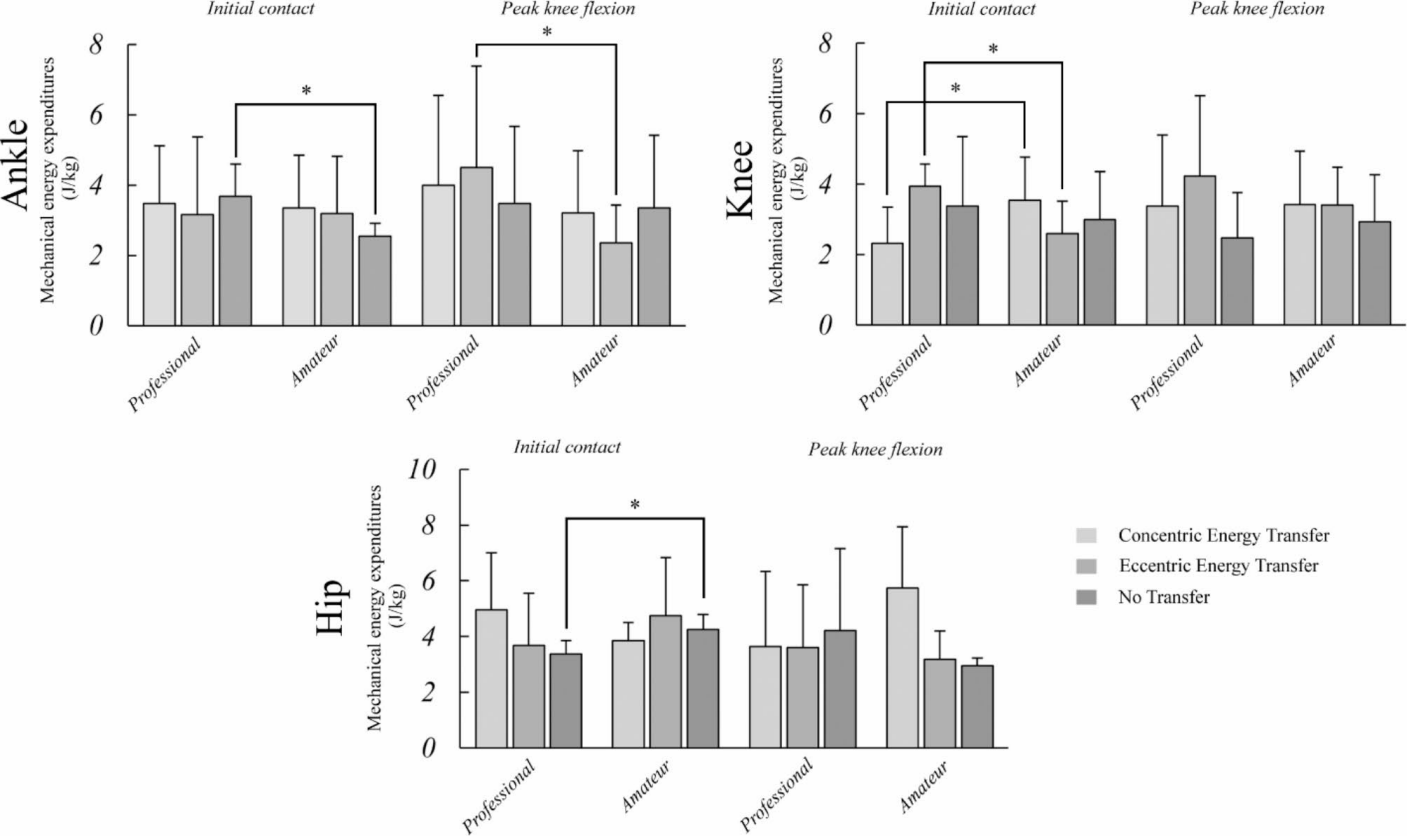
An illustration to demonstrate statistically significant differences that were observed between professional and amateur athletes

Regarding mechanical energy transfer, the independent t-test demonstrated significant differences between ankle MEEn, knee MEEc, knee MEEe, and hip MEEn in initial heel contact. At the same time, there was only a statistically significant difference between ankles MEEe in maximal knee flexion angle (Table 2). Furthermore, analyzing mechanical energy compensation coefficients (MECs) showed that there were significant differences between two groups in ankle MECc, knee MECc, knee MECe, and hip MECe at the initial contact phase and between ankle MECe, knee MECc, knee MECe, hip MECc, and hip MECe at maximal knee flexion angle (Table 3). Figure 3 illustrates the differences exist among professional and amateur athletes at initial contact and maximal knee flexion angle time points.

**Table 2:** Mechanical energy expenditures (MEEs) (J/kg) of the ankle, knee and hip during the Initial contact and the Peak knee flexion of front and right lunging (Mean ± SD) for each transfer condition (concentric transfer, eccentric transfer, and no transfer)

Variable		Initial contact				Peak knee flexion		
		Professionals		Amateur	p-Value^†^	Professionals	Amateur	p-Value
		Mean ± SD				Mean ± SD		
Ankle	MEE_C_	3.47 ± 1.64	3.34 ± 1.51		0.249	4.00 ± 2.54	3.21 ± 1.76	0.433
	MEE_E_	3.15 ± 2.22	3.19 ± 1.62		0.161	4.49 ± 2.89	2.35 ± 1.08	< 0.042^*^
	MEE_N_	3.68 ± 0.91	2.54 ± 0.37		< 0.003^*^	3.47 ± 2.19	3.34 ± 2.07	0.495
Knee	MEE_C_	2.32 ± 1.02	3.54 ± 1.22		< 0.026^*^	3.38 ± 2.01	3.42 ± 1.52	0.260
	MEE_E_	3.94 ± 0.63	2.59 ± 0.92		< 0.001^*^	4.23 ± 2.28	3.41 ± 1.06	0.318
	MEE_N_	3.37 ± 1.97	2.99 ± 1.37		0.624	2.48 ± 1.27	2.93 ± 1.33	0.449
Hip	MEE_C_	4.95 ± 2.06	3.86 ± 0.64		0.138	3.65 ± 2.68	5.74 ± 2.19	0.072
	MEE_E_	3.64 ± 1.88	4.74 ± 2.09		0.232	3.61 ± 2.25	3.18 ± 1.02	0.585
	MEE_N_	3.38 ± 0.47	4.25 ± 0.54		< 0.001^*^	4.22 ± 2.93	2.96 ± 0.26	0.193

**Note**: *statistically significant differences between the professionals and Amateur groups. **P-value < 0.05 is considered to be statistically significant; MEC always zero for no-transfer conditions

**Abbreviations:** MEC_C_, concentric energy transfer condition; MEC_E_, eccentric energy transfer condition

**Table 3:** Mechanical energy compensation coefficients (MECs) of the ankle, knee, and Initial hip contact and the Peak knee flexion of front and right lunging (Mean ± SD) for concentric and eccentric energy transfers

Variable		Initial contact			Peak knee flexion		
		Professional	Amateur	p-Value	Professional	Amateur	p-Value
		Mean ± SD			Mean ± SD		
Ankle	MEC_C_	0.42 ± 0.07	1.69 ± 0.16	< 0.001^*^	0.52 ± 0.08	0.44 ± 0.24	0.318
	MEC_E_	0.88 ± 0.06	0.84 ± 0.08	0.302	0.79 ± 0.13	0.61 ± 0.13	< 0.007^*^
Knee	MEC_C_	1.71 ± 0.57	0.29 ± 0.28	< 0.001^*^	0.03 ± 0.02	0.64 ± 0.16	< 0.001^*^
	MEC_E_	1.81 ± 0.57	0.44 ± 0.32	< 0.001^*^	0.30 ± 0.06	2.04 ± 0.33	< 0.001^*^
Hip	MEC_C_	1.48 ± 0.20	1.33 ± 0.27	0.198	1.46 ± 0.51	0.38 ± 0.23	< 0.001^*^
	MEC_E_	2.46 ± 1.21	0.38 ± 0.10	< 0.001^*^	1.37 ± 0.10	0.32 ± 0.16	< 0.001^*^

**Note:** *statistically significant differences between the Professional and Amateur groups. **P-value < 0.05 is considered to be statistically significant; MEC always zero for no-transfer conditions

**Abbreviations:** MEE_C_, concentric energy transfer condition; MEE_E_, eccentric energy transfer condition; MEE_N_, no transfer condition

## Discussion

The main objective of this study was to compare the mechanical energy transfers attributable to right-forward lunges between amateur and professional badminton players. The results showed that the professional players demonstrated statistically significant more ankle no-transfer, less knee concentric transfer, more knee eccentric transfer, and less hip no-transfer at the initial heel contact phase. At the same time, the amateur athletes showed significantly more ankle eccentric transfer at maximal knee flexion angle time point. Analyzing mechanical energy compensation coefficients showed that the professional athletes had significantly less ankle concentric transfer, more knee concentric transfer, more knee eccentric transfer, and more hip eccentric transfer at initial contact phase. While they found to have significantly more ankle eccentric transfer, less knee concentric transfer, less knee eccentric transfer, more hip concentric transfer, and more hip eccentric transfer at maximal knee flexion angle.

Study results showed that professional and amateur badminton players had different mechanical energy transfers in initial heel contact in ankle MEEn, knee MEEc, knee MEEe, and hip MEEn. However, there were only significant differences between ankles MEEe in maximal knee flexion angle. In the ankle no transfer condition, the average mechanical energy expenditure was lower in amateur athletes, whereas in the hip no transfer condition, it was higher in amateur athletes than in professional athletes at the initial heel contact. This finding is in line with previous research suggesting that amateur athletes may employ different movement strategies during their performance [10, 17, 32]. While this study is the first to examine energy flow in both professional and amateur badminton players, its findings are consistent with other biomechanical studies conducted in this area [9, 10, 12, 13]. The biomechanical properties of the badminton lunging step have been studied in previous reports [9, 10, 12, 13, 33]. For instance, some studies demonstrated that the kinematics of the hip, knee, and ankle [12], power of joints, foot impulse [13], and peak plantar pressures [9, 10, 33] differed significantly between professional and amateur badminton athletes while performing a right-forward lunging step with backhand shot.

The increase in concentric mechanical energy expenditures in the knee joint indicated that knee muscles generated greater energy in amateur players, while the increase in eccentric mechanical energy expenditures in professional athletes indicated that they had higher energy absorption in the knee segments [18, 29, 30]. On the other hand, it was observed that at the initial heel contact, the amount of no transfer mechanical energy expenditure increased in the hips of amateur athletes and decreased in their ankles. This finding suggests that the amount of energy not absorbed by the knee joint in amateur athletes is more likely to be transferred to their ankles. Therefore, it appears that the risk of knee and ankle injuries may be higher in amateur athletes than in professional athletes [18, 29, 30].

One possible explanation for these findings is that the observed changes may be the result of mechanical alterations in adjacent joints [3], such as a shift in the energy production of the hip or ankle muscles. Accordingly, earlier research suggested that amateur athletes might have varied muscle motion ranges and functions during lunge movements [3, 8–11]. As an illustration, Huang et al. (2014) discovered that badminton players with a history of knee injuries purposefully adopted a larger knee flexion to protect the joint during the landing [10]. When performing a forward lunge, the activity of the quadriceps femoris, biceps femoris, and gastrocnemius muscles was associated with knee and hip motion in the sagittal plane [10, 33]. Also, this change in energy expenditure may cause uncoordinated movements, which may predispose the athletes to the risk of suffering more injuries [22].

Concerning MECs, significant differences were observed between two groups in ankle MECc, knee MECc, knee MECe, and hip MECe at the initial contact phase and between ankle MECe, knee MECc, knee MECe, hip MECc, and hip MECe at maximal knee flexion angle. The ratio of muscle energy that was compensated by inter-segmental energy transfer in initial heel contact was less for the ankle muscles in professional players. While for amateur players, this ratio at the hip and knee muscles was significantly reduced at initial heel contact. Professional players seemed to transfer energy across the knee and ankle distally at initial heel contact, whereas amateur players transferred lesser energy across the knee. Compensation coefficients suggest that professional players probably demonstrated more energy transfer in lower extremity segments compared to the energy expended by muscles (especially at the knee) [9, 10]. Conversely, the amateur players showed excess energy transfer towards the pelvis during a lunge, which is probably being utilized to advance the contralateral leg. Overall, it appears that amateur players had less energy transfer in their knee and hip joints, which may lead to more energy being absorbed by their muscles and potentially increase their risk of muscular injuries.

Also, badminton players in this study showed that lower extremity muscles require more muscle work to produce similar power output, that these differences were more evident at maximal knee flexion angle than initial heel contact moment. In professional players, the energy compensation associated with ankle, knee, and hip muscles was significantly higher.

Overall, based on the aforementioned points, it seems that amateur athletes are at a greater risk of knee and ankle injuries compared to professional athletes, and they are also more likely to experience muscular injuries in their lower extremities. Due to the increased risk of injury when performing a right-forward lunge, it is recommended for amateur players to follow a comprehensive training program that promotes proper lunging technique [34].

### Limitations

Various limitations should be considered, given the main findings of this study. Only college-aged females were included in this study, and we did not take a more extensive age range for either gender into account. Moreover, it is suggested to conduct another study for men players to clarify the issue. On the other hand, the study participants had no musculoskeletal disorders, so athletes with musculoskeletal disorders like low back pain or knee injuries may present different mechanical energy transfer mechanisms. Moreover, another limitation is that in this study the athletes examined just when players are out of a fatigued state. When playing, it is not known what fatigue influence and results. Other study limitation may arise from the fact that in the current study the forward lunge movement examined in the laboratory setting that may be not similar to players’ on-court movements and contextual variables in badminton. So our findings may not be generalizable to on-court forward lunge or movements.

## Conclusion

In conclusion, we have shown that the mechanical energy efficiency of the right-forward lunge is skill-related. It seems that altered lunge landing biomechanics may increase the risk of ankle and knee injuries and muscular damages in amateur athletes. It is recommended for amateur players to follow a injury prevention training program that promotes proper lunging technique.

### Electronic supplementary material

Below is the link to the electronic supplementary material.

Supplementary Material 1

## Data Availability

The datasets generated and analysed during the current study are available in the supplementary file 1 in the journal.
